# Exploring Feature Dimensions to Learn a New Policy in an Uninformed Reinforcement Learning Task

**DOI:** 10.1038/s41598-017-17687-2

**Published:** 2017-12-15

**Authors:** Oh-hyeon Choung, Sang Wan Lee, Yong Jeong

**Affiliations:** 1 Department of Bio and Brain Engineering, Korea Advanced Institute of Science and Technology, 34141 Daejeon, Republic of Korea; 2 KI for Health Science and Technology, Korea Advanced Institute of Science and Technology, 34141 Daejeon, Republic of Korea; 3 Program of Brain and Cognitive engineering, Korea Advanced Institute of Science and Technology, 34141 Daejeon, Republic of Korea; 4 KI for Artificial Intelligence, Korea Advanced Institute of Science and Technology, 34141 Daejeon, Republic of Korea; 5 Present Address: Laboratory of Psychophysics, Brain Mind Institute, École Polytechnique Fédérale de Lausanne (EPFL), 1015 Lausanne, Switzerland

## Abstract

When making a choice with limited information, we explore new features through trial-and-error to learn how they are related. However, few studies have investigated exploratory behaviour when information is limited. In this study, we address, at both the behavioural and neural level, how, when, and why humans explore new feature dimensions to learn a new policy for choosing a state-space. We designed a novel multi-dimensional reinforcement learning task to encourage participants to explore and learn new features, then used a reinforcement learning algorithm to model policy exploration and learning behaviour. Our results provide the first evidence that, when humans explore new feature dimensions, their values are transferred from the previous policy to the new online (active) policy, as opposed to being learned from scratch. We further demonstrated that exploration may be regulated by the level of cognitive ambiguity, and that this process might be controlled by the frontopolar cortex. This opens up new possibilities of further understanding how humans explore new features in an open-space with limited information.

## Introduction

Difficulties in decision-making arise when we are bombarded with information, but cannot determine which pieces of information are relevant to the situation. Learning to achieve better outcomes requires exploring new information and integrating related information. For instance, baseball players must decide whether to swing the bat (i.e., “go” or “no go”) based on their observation of the pitcher’s throwing motion, which is composed of several characteristics (“features”). When first confronting the pitcher, the batter does not know which features are useful, though he or she explores new features with each pitch, gradually learning how they are related. Thus, both *exploring* new features and *learning* the relationships among these features are necessary for making a decision when presented with incomplete information.

Several studies have investigated exploratory behaviours and decision-making in simple situations. Such studies have often examined exploration-exploitation scenarios, in which participants must choose whether to try a new action (exploration), stick to a known action (exploitation)^1,2^, or use a new hypothetical state-space^3^. Although, Schuck *et al*. investigated the use of alternative strategies during exploration behaviour (using colours instead of corners) and the corresponding changes in neural activity when participants were provided with incomplete information^4^, the process by which humans explore additional features within a multi-dimensional environment when making decisions based on incomplete information remains to be fully understood. Previous studies involving weather-prediction tasks have revealed that humans can utilise various strategies to make a decision based on several cues^5^. Additionally, some studies have examined multi-dimensional decision-making, but detailed, complete explanations of the task structure were provided to the participants in all such studies. Furthermore, most of these studies have focused mainly on a fixed set of features (e.g., dimensional reduction^6^ and feature attention^7^), or on competition among features^8^. In addition, although evidence regarding the transfer of learned characteristics to novel situations (transfer learning) from perceptual learning studies remains controversial, few studies have investigated value learning processes in the context of increasing features^9^.

The neural substrates for exploration and learning processes have been well-studied in the last decades. Values for each stimulus of an event are widely represented in ventromedial prefrontal cortex (vmPFC)^1,10–12^ and ventral striatum (VS)^12,13^. Also, motion-related values are known to be encoded in intraparietal sulcus (IPS)^1,3^. In addition, dorsal striatal areas such as the putamen^11,14^ are accepted as reward prediction error-encoding areas in the more automatic and habitual learning situations. Finally, frontopolar cortex (FPC) is largely activated in various exploration states, including alternative actions^1,2^ and alternative rules^15^. Therefore, we wanted to examine whether or not these common areas are correspondingly activated in our task.

In the present study, we utilised computational models and functional magnetic resonance imaging (fMRI) to investigate the process by which humans explore and learn new features for improving decision-making outcomes when information about these features has not been provided. To examine exploratory and learning behaviour in a multi-dimensional environment, we designed and conducted a novel multi-dimensional reinforcement learning task in which fixed rewards were associated with relationships among three distinct dimensional features (shape, colour, and pattern) without any instructions.

Exploration and learning behaviours were modelled by a combination of two computational models: A probabilistic policy exploration model and a value transfer learning model. These computational models were applied under the assumptions that 1) exploratory behaviour can be observed by tracking the use of current policy, and 2) learning of new information would develop by adopting previously learned values. Behaviour-related brain activities were assessed by fMRI analysis within previously defined regions of interest using the model parameters.

## Results

### Multi-dimensional reinforcement learning task

**Figure 1:**
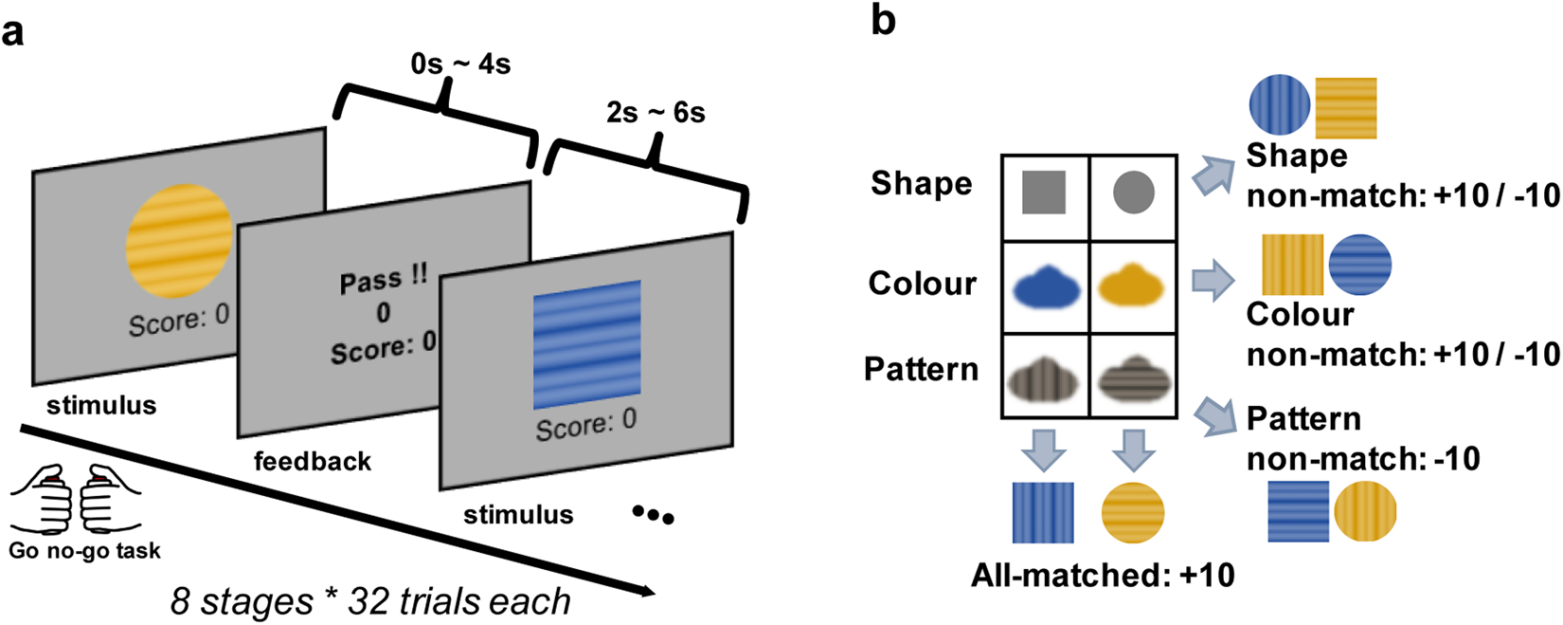
Behavioural task design. (**a**) Schematic of multi-dimensional reward learning task. In each trial participants were presented with a single visual stimulus composed of three different dimensional features: shape, colour, and pattern. When each stimulus image was presented, participants were asked to make a selection by pressing the left or right button within 4 s, following which feedback was provided for a duration of 2–6 s. A total of 256 trials were presented to each participant in random order. (**b**) Feature dimensions of visual stimuli. When chosen images are one of the two rewarded stimuli (“all-matched” combinations) – the blue-square-vertical and the yellow-circle-horizontal patterned images – participants received +10 points. When “pattern non-match” images were selected, participants received −10 points. All other combinations (“shape non-match” and “colour non-match” images) were randomly associated with +10 or −10 points. The last two combinations were added to adjust task difficulty.

To encourage exploration of new features, we designed a multi-dimensional reinforcement learning task in which no information about the task structure was provided to the participants. Twenty-nine adult participants (12 females; age range, 20–29 years; mean age, 22.4 ± 2.27; all subjects were right-handed) performed the task during fMRI scanning. The stimuli was consisted of three-dimensional features including shape, colour, and pattern (Fig. 1). Participants were only instructed to gather as many points as possible, requiring them to acquire dimensional information by trial-and-error. However, unbeknownst to participants, a fixed rule was applied to each stimulus: Points were awarded only for the two all-match cases (blue-square-vertical and yellow-circle-horizontal patterned figures), while points were lost in the two pattern non-match cases (blue-square-horizontal and yellow-circle-vertical patterned figures). In all other situations, points were awarded or lost at a rate of 50% each (see *Methods: Behavioural task* for further details). Prior to the actual behavioural task, we performed a simulation to verify that an optimal strategy for this task is to use all three-dimensional information (i.e., features; see *Supp. Methods: Policy simulation* for further details). The results of this simulation revealed significantly higher performance when all three-dimensional features were used (S1 Fig. 1a,b).

Most participants (86%, 25/29) performed superior to the chance level of performance (the chance level, p < 0.05, cut-off score 156 points). Brief questionnaires administered following completion of the task revealed that participants tended to integrate dimensional components gradually. However, four participants reported that they were unable to learn any useful information and thus performed randomly.

**Figure 2:**
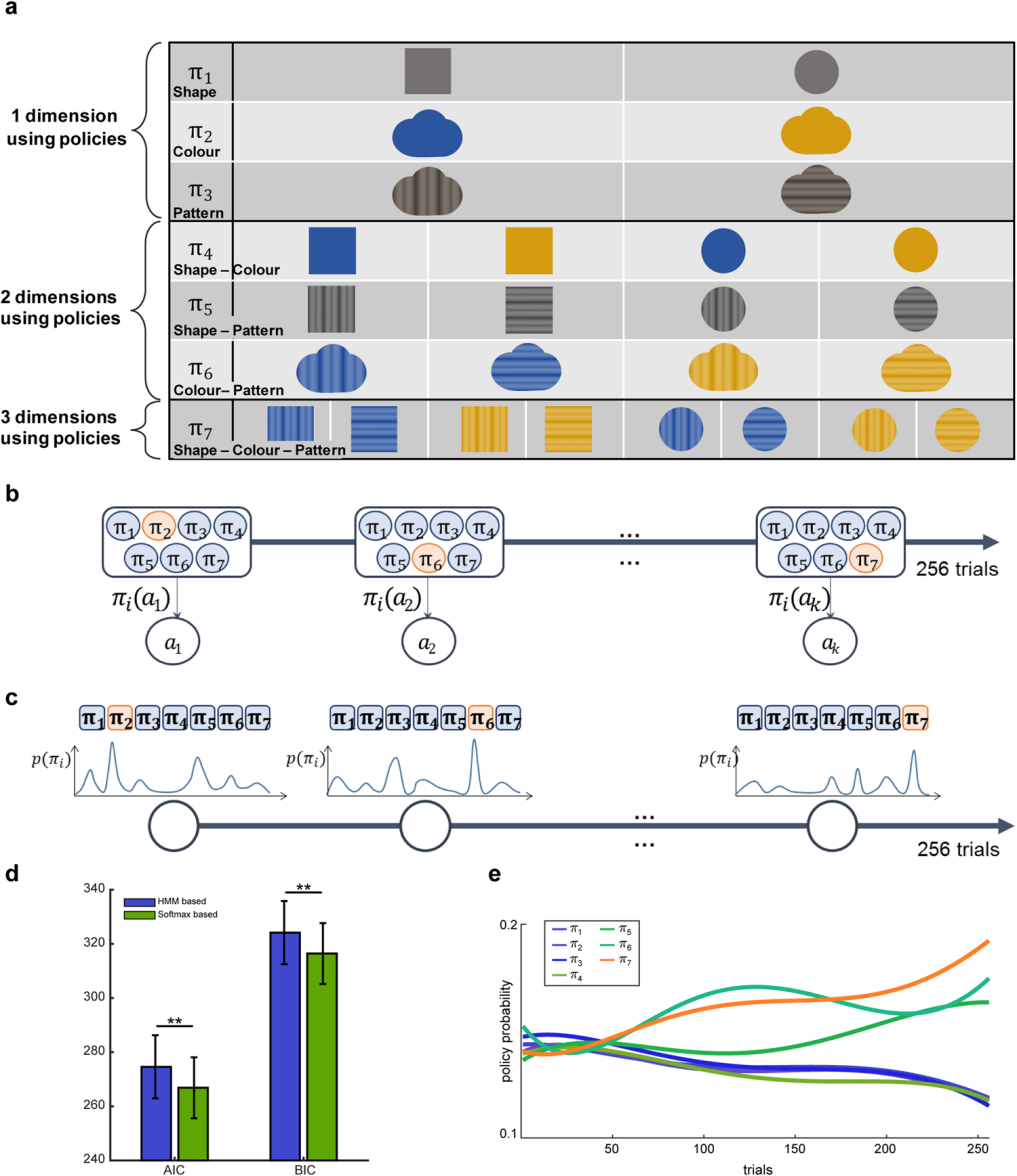
Probabilistic policy exploration model. (**a**) In the naïve Reinforcement Learning (RL) phase, possibly used features were abstracted as policies, as follows: π_1_, using shape information (1 dim), π_2_, using colour information (1 dim), π_3_, using pattern information (1 dim), π_4_, using combinations of colour and shape information (2 dim), π_5_, using combinations of shape and pattern information (2 dim), π_6_, using combinations of colour and pattern information (2 dim), π_7_, using combinations of shape, colour, and pattern information (3 dim). (**b**) A schematic diagram of the hidden Markov model (HMM)-based policy search model. (**c**) A schematic diagram of the softmax function-based policy search model. (**d**) Comparison of model results. blue: HMM-based model, green: Softmax function-based policy search model, paired t-test p = 0.0080, mean ± SEM. (**e**) Representative fitted policy probability. Each policy is represented by an individual colour.

**Figure 3:**
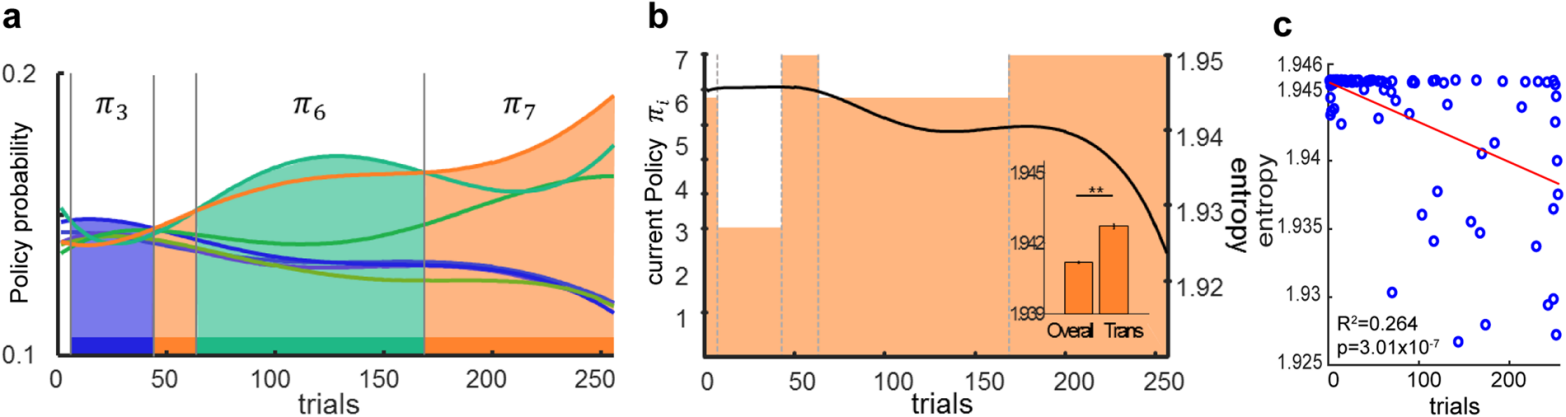
Representative policy estimation and corresponding entropy. (**a**) Policy estimation. The policy with the highest probability estimate in each trial was regarded as a currently used policy. (**b**) Current policy within each trial (orange squares, grey dot: time-points that policy transition occurred) and policy entropy values (black line). Difference in entropy values between policy transition time-points and all the other trials (inset, paired t-test, p < 0.01, mean ± SEM). (**c**) Transition time-point with regard to entropy and trial order. (Blue dot: each transition time-points for all participants, red line: linear regression result.) Transition time-points were significantly related to earlier trials and higher entropy (R^2^ = 0.264, p = 3.01 × 10^−7^).

**Figure 4:**
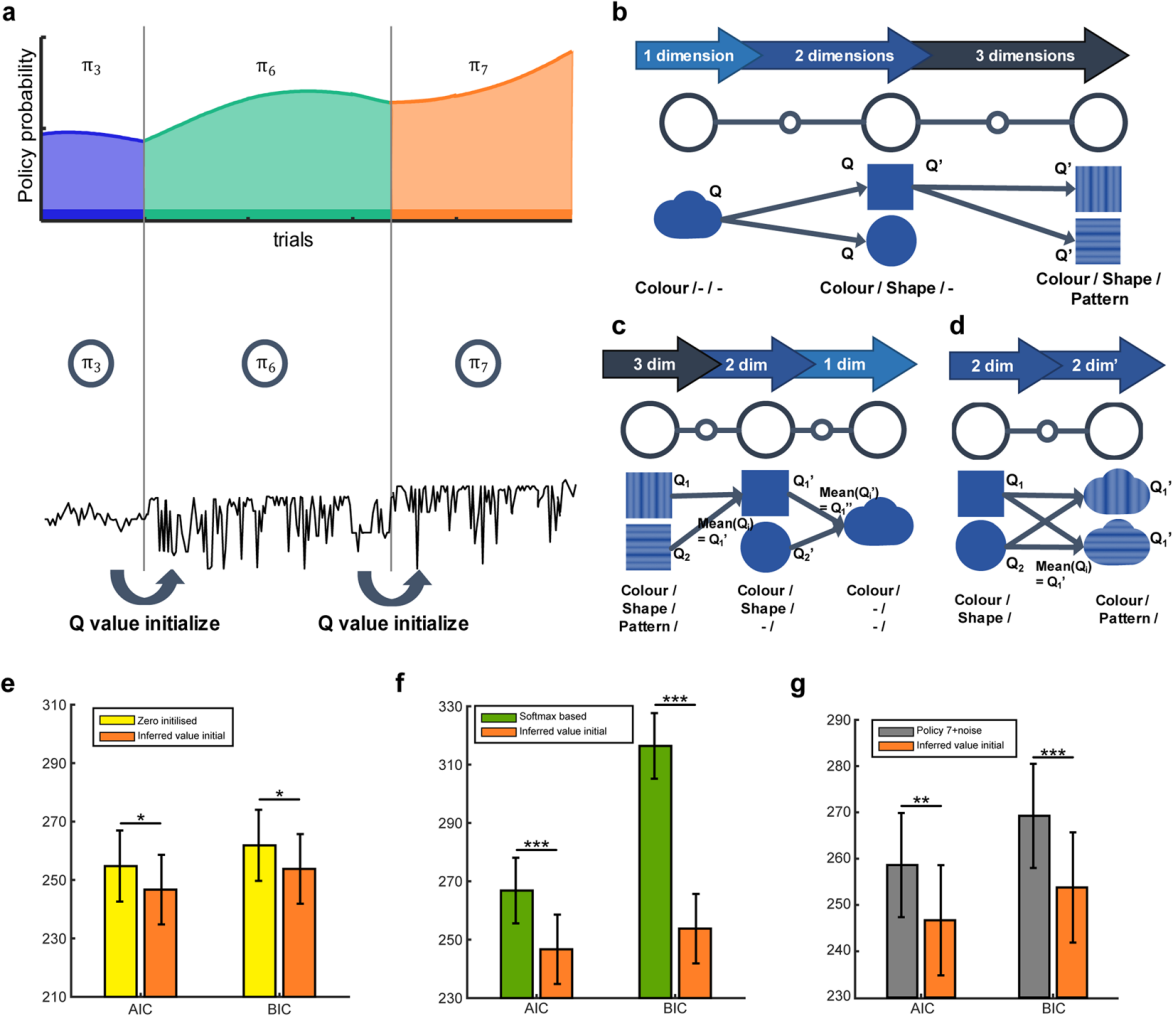
Value Transfer Learning Model. (**a**) Value transfer learning model with policy changes. (**b**,**c**,**d**) Learning based on previously learned state-action values, (**b**) Increasing feature dimensionality case, (**c**) Decreasing feature dimensionality case, (**d**) Policy transition without a change in feature dimensionality. (**e**) Model comparison between the zero initialised and learned value initialised model (paired t-test, mean ± SEM, *p < 0.05). (**f**) Model comparison between softmax function-based policy search model and inferred value transfer learning model (paired t-test, mean ± SEM, **p < 0.01, ***p < 0.001). (**g**) Model comparison between policy seven with noise model and learned value initialised model (paired t-test, mean ± SEM). Yellow, zero initialised model; orange, learned value initialised model; green, sofmax function-based policy search model; grey, policy seven with noise model.

In addition to the fMRI experiment, another 29 participants (11 females; age range, 20–29 years; all subjects were right-handed) performed an additional behavioural task: Rather than using a single rule for penalizing stimuli, 10 participants performed the pattern non-matched task (same as the main task), another 10 performed the shape non-matched task, and the remaining nine performed the colour non-matched task (S2 Fig. 2). The results of these tasks were similar to those of the main task, confirming that learning performance did not depend on the types of features (S3 Fig. 3, S4 Fig. 4).

### Probabilistic policy exploration model

Based on responses obtained via a post-experiment questionnaire, the majority of participants reported that they tended to gradually integrate task-relevant information regarding feature dimensions. A gradual increase in task performance was also observed (S6 Fig. 6). To more closely examine participants’ feature exploratory behaviour, we utilised a two-step probabilistic policy exploration model as below. First, we considered a computational hypothesis that subjects develop policies for state-spaces that are composed of each feature level, and then estimated how they take certain action under each state-space using naïve reinforcement learning (RL). Estimating the policy for choosing a state-space using the two-policy exploration models enabled us to identify which features were used in each trial.

First, we considered all possible combinations of available features (shape, colour, pattern), which constituted seven different policies (Fig. 2a). Assuming that participants would use all seven policies, learning behaviours were modelled using a naïve RL algorithm (the Rescorla-Wagner model, equation (1))^16,17^, which updates values by adding weighted reward prediction errors. Therefore, we examined the seven sets of trial-by-trial state-action values for each participant (Fig. 2a).

1 $${{\rm{Q}}}_{{{\rm{a}}}_{{\rm{\pi }}i}}^{{\rm{new}}}({S}_{stimulus})={{\rm{Q}}}_{{{\rm{a}}}_{{\rm{\pi }}i}}^{{\rm{old}}}({S}_{stimulus})+\alpha ({R}_{t}-{{\rm{Q}}}_{{{\rm{a}}}_{{\rm{\pi }}i}}^{{\rm{old}}}({S}_{stimulus})),{\rm{for}}\,i\in [1,7]$$

*Q* represents the state-action value of the stimulus, *π*_*i*_ represents the behavioural rule using a state-space *i*, *R*_*t*_ represents the reward for each trial, and *α* represents the free parameter learning rate, which refers to the weighted factor associated with the reward prediction error (RPE).

2 $${{\rm{\pi }}}_{{\rm{i}}}({{\rm{a}}}_{{\rm{k}}})=\frac{{e}^{\beta {Q}_{{a}_{k}}{\rm{\pi }}i}}{{\sum }_{j=1}^{2}{e}^{\beta {Q}_{{a}_{j}}{\rm{\pi }}i}},{a}_{k}=[0,1]\,{\rm{for}}\,i\in [1,7]$$

In this case, *β* represents the inversed temperature parameter, which regulates the steepness of the function. The choice probability (selection of either the right or left button) was then calculated using the *softmax* function (equation (2))^1^. Here, prior probabilities for each choice and policy were fixed and stable.

To identify the policy used by participants in each trial, we implemented two computational models, the hidden Markov Model (HMM)-based^18–21^ policy search model, and the *softmax* function-based policy search model. The HMM-based method infers seven policy probabilities for the current trial based on probabilities from the previous trial. In contrast, the *softmax* function-based model assumes that the seven policy probabilities are temporally independent. Probabilities of the seven policies in each trial were estimated using the two models (Fig. 2e). The policy associated with the highest polynomial fitted probability was regarded as the one currently used by the participant (Fig. 3a). We then computed model likelihoods using the choice probability given by the model (equation (3)).

3 $${\rm{L}}={\rm{p}}({C}_{1:{\rm{T}}}{|{\rm{\Theta }}}_{{\rm{m}}}\,)=\,\prod _{t=1}^{T}p({C}_{t}|{D}_{1:t-1},{{\rm{\Theta }}}_{m})$$

Here, *C*_1:T_ represents each participant’s behavioural data, while *Θ*_*m*_ represents the free parameters of the model (i.e., learning rate *α*, or inverse temperature parameter *β*).

We fitted the parameters of each model to the behavioural data by maximising the log-likelihood (equation (3)), and then computing both Akaike information criterion (AIC)^22^ and Bayesian information criterion (BIC)^23^ scores (see *Supp. Methods: Model comparison*). We observed that the *softmax* function-based policy search model best explained participant behaviour (AIC, BIC, p = 0.0080, paired t-test) (Table 1, Fig. 2d). Therefore, we used the *softmax* function-based policy search model to identify behavioural strategies and classify them into four categories: 1) increasing the feature dimensionality from two to three (2d to 3d), 2) reducing feature dimensionality from three to two (3d to 2d), 3) using all three-dimensional features throughout the experiment (3d-only), and 4) using two dimensional features throughout the experiment (2d-only). The majority of subjects (15/29) were observed to increase feature dimensionality by exploring new features, though some were observed to reduce the feature dimensionality (4/29), and some did not explore new features (2d-only group: 2/29; 3d-only group: 4/29). There were no significant differences in performance among these four groups (S6 Fig. 6a). As expected, the four participants who reported that they were unable to learn useful information during the experiment did not belong to any of these groups, but instead tended to use only one-dimensional feature.

**Table 1:** Overall model results, model comparison, and fitted parametric values.

	Model Comparison				Model parameters	
	Log-likelihood	AIC	BIC	# of param	α	β
Learned val ini	−121.35 ± 32.05	246.70 ± 64.10	253.79 ± 64.10	2	0.25 ± 0.25	4.47 ± 5.19
Zero ini	−125.38 ± 32.79	254.77 ± 65.58	261.86 ± 65.58	2	0.22 ± 0.25	4.30 ± 2.83
Naïve + Softmax	−119.42 ± 30.30	266.85 ± 60.59	316.43 ± 60.59	14	0.22 ± 0.28	10.26 ± 19.25
Naïve + HMM	−123.30 ± 31.38	274.59 ± 62.77	324.17 ± 62.77	14	0.21 ± 0.26	14.69 ± 38.55

Table of overall models, model comparison and fitted parametric values. Learned val ini: Value transfer learning model initialised with learned value. Zero ini: Value transfer learning model initialised with zero. Naïve + Softmax: Probabilistic policy exploration model with softmax function-based policy search. Naïve + HMM: Probabilistic policy exploration model with HMM based policy search. Log-likelihood value: Larger values indicate a better fit. AIC and BIC: Smaller values indicate a better fit. All values are represented as mean ± SD, except number of parameters. HMM: hidden Markov model; AIC: Akaike information criterion; BIC: Bayesian information criterion.

### Value transfer learning model

The *softmax* function-based policy search model provides trial-by-trial estimates of policy probabilities. We used predictions of this model to identify policies adopted by each participant. Specifically, the policy associated with the highest polynomial fitted probability in each trial was regarded as the one currently in use (Fig. 3a)^24,25^. We then identified trials in which policy transition occurred (Fig. 3b, grey dotted line).

Learning occurred in each identified current policy, and was shifted to another policy in each transition time-point. Although the previous model provided information regarding when policy transitions occur, how state-action values (*Q-*values) are initialised at each transition remained unclear. Increasing behavioural and computational evidence from perceptual learning^9^ and machine learning studies^26,27^, respectively, has suggested that the initial value of new features can be inferred from previously learned values. To verify this hypothesis, we compared the computational model, which initialises *Q-*values based on previously learned values, with the zero-initialised model.

The value learning process was nearly identical to that of the previous naïve reinforcement learning model (equation (1)), except that only one identified policy was used for each trial. Therefore, the state-action value of the new policy was updated at the policy transition time-points based on one of the two initialising rules.

In the first scenario, Q-values for the upcoming policy were initialised to zero. This model assumes that all values are re-learned when participants change their decision-making policy.

4 $${{\rm{Q}}}_{{{\rm{a}}}_{{\rm{\pi }}\mathrm{curr}}}^{{\rm{new}}}({S}_{stimulu{s}_{type}})=0,\,\forall \,type$$

In the second scenario, *Q-*values for the upcoming policy were initialised as values inferred from previous policies. This model assumes that, even when the policy has changed, learned values are not removed, but are instead reused for further learning processes, suggesting that initialisation of values using previously learned values is associated with better performance.

$$\mathrm{in}\,\mathrm{the}\,\mathrm{case}\,::\mathrm{dimension}\,\mathrm{increase}$$

$${{\rm{Q}}}_{{{\rm{a}}}_{{\rm{\pi }}{\rm{curr}}}}^{{\rm{new}}}({S}_{stimulu{s}_{type}})={{\rm{Q}}}_{{{\rm{a}}}_{{\rm{\pi }}pre}}^{{\rm{old}}}({S}_{stimulu{s}_{type}}),\forall \,type$$

$$\mathrm{in}\,\mathrm{the}\,\mathrm{case}\,::\,\mathrm{dimension}\,\mathrm{decrease}\,||\,\#\,\mathrm{of}\,\mathrm{dimensions}\,\mathrm{unchanged}$$

5 $${{\rm{Q}}}_{{{\rm{a}}}_{{\rm{\pi }}curr}}^{{\rm{new}}}({S}_{stimulu{s}_{type}})={\rm{mean}}({{\rm{Q}}}_{{{\rm{a}}}_{{\rm{\pi }}pre}}^{{\rm{old}}}({S}_{stimulu{s}_{type}})),\forall type$$

In the present study, learned Q-values were directly utilised in the case of increasing feature dimensionality, while the mean of several learned Q-values – which form a subpart of the new policy – were utilised in the case of decreasing or unchanged feature dimensionality (Fig. 4c,d). In the latter cases, the mean value was used because each Q-value reflects the overall exposure of related features, as well as some noise associated with values from non-related features. No additional weighting method is needed. Here, two free parameters – learning rate α and inverse temperature parameter β – were independently fitted to each participant’s behavioural data.

When using AIC and BIC criteria, we observed that the second scenario – which infers the value based on that for the previously used policy – best explained participant behaviour (AIC, BIC, p < 0.05, paired t-test) (Fig. 4e, orange). This result supports the hypothesis that, when participants begin to learn values of a novel state or feature, they utilise value information of the relevant stimuli that has been previously learned, as opposed to “learning from scratch”.

### Exploration behaviour in an uninformed multi-dimensional reinforcement learning task

We first examined feature exploratory behaviour using a probabilistic policy exploration model. However, the probabilistic policy exploration model is not a realistic model, as it assumes all seven policies are processed simultaneously. To fully address this issue, we tested the value transfer learning model that chooses the policy with the highest policy probability, which is a near-optimal model. We observed that this near-optimal learning strategy best accounted for the behaviour of participants in the present study (AIC and BIC, ValTrans with inferred val model > Naïve + Softmax, paired t-test, p < 0.001) (Fig. 4f). Furthermore, our near-optimal learning strategy even better explained the use of all three dimensions with random noise (ɛ) (AIC and BIC, ValTrans with inferred val model > Policy 7 + ɛ, paired t-test, **p < 0.01, ***p < 0.001) (Fig. 4g). Overall results for the model comparison are shown in Table 1 (additional model comparisons are in the *Supplementary text*).

To further address the question of which factor mediates the exploration of features, we computed policy entropy as follows:

6 $${\rm{H}}({\rm{\pi }})=-\sum _{i=1}^{7}P({\pi }_{i})logP({\pi }_{i})$$

When probabilities of each policy become similar, the entropy H(π) increases. This refers to the situation in which participants become confused about which policy they choose to obtain the desired outcome. That is, cognitive ambiguity increases. We observed a tendency for more frequent transitions in early trials, during which policy entropy is high (Fig. 3c). Also, entropy at transition time-points was significantly higher than the entropy over all time points (256 trials) (Fig. 3b inset, t-test, p < 0.01). These results indicate that the exploration of new information occurs when cognitive ambiguity is high.

We also observed a significant, positive linear correlation between our suggested model—which explores new features under high cognitive ambiguity and learns based on previously learned values—and participants’ performance (R^2^ = 0.7137, p = 8.3 * 10^−9^) (S7 Fig. 7). Specifically, a linear correlation was observed between the likelihood of the model and the behavioural task score (task performance). This result suggests that cognitive ambiguity-driven exploration and value transfer learning occur during multi-dimensional decision-making, and that these processes underlie the development of new learning algorithms.

### Neural correlates of policy exploration

To examine the neural computation underlying policy exploration, we regressed signals of the computational model against the fMRI data. The general linear model (GLM) consisted of the following parametric regressors: state-action value signals, prediction error signals from the value transfer learning model, entropy as a cognitive ambiguity signal, and transition time-points from the softmax function-based exploration model as measures of a conducted exploration signal, as well as stimuli time-points, feedback time-points, and response times. We performed an ROI analysis as follows: (1) defining masks of all of the ROIs, and then (2) using the combined masks as our small volume correction (SVC) mask. The ROIs are defined as a sphere of 10 mm radius around the predefined coordinates, including ventromedial prefrontal cortex (vmPFC) [−3 33 −6]^1^ and right ventral striatum (VS) [6 8–4]^13^ for value signals; left and right putamen [left: −24 6 9, right: 27–13 10]^11^ for reward prediction error signals; and frontopolar cortex (FPC) [18 65 10]^15^ for exploration signals (detailed descriptions of ROIs are in S11 Table 3). All were survived after SVC (voxel-level correction p < 0.05; Table 2). In addition, the results survived from whole-brain family-wise error correction for multiple comparisons (Bonferroni correction, p < 0.05, S4 Table 4) were reported. In order to investigate the neural substrates regarding when a person is seeking a new feature dimension, we analysed only the fMRI data of the feature dimension integrating groups (2d to 3d; n = 13/23, excluding the four non-learners and two participants with excessive head motion).

**Table 2:** Parametric regression of brain areas associated with value signals, error signals, entropy, and transition time-points.

Regressors		MNI Coordinate				Statistics		
		x	y	z	voxel #	Corrections	T	P_corr_
Value	rVS	9	12	−9	61	SVC [6 8 −4]^13^	5.80	p = 0.0126
	vmPFC	0	33	−9	38	SVC [−3 33 −6]^1^	5.24	p = 0.0309
Error	lPutamen	−21	9	9	74	SVC [−24 6 9]^11^	5.33	p = 0.0011
	rPutamen	27	−15	9	58	SVC [27 −13 10]^11^	7.49	p = 0.0280
Entropy	FPC	12	60	3	20	SVC [18 65 10]^15^	4.17	p = 0.0455

SVC: small volume correction, all volumes were derived from previously published studies, cited respectively. Only clusters with p_corr_ <0.05 and cluster-size >10 voxels were reported, in SVC with a 10 mm radius sphere.

**Figure 5:**
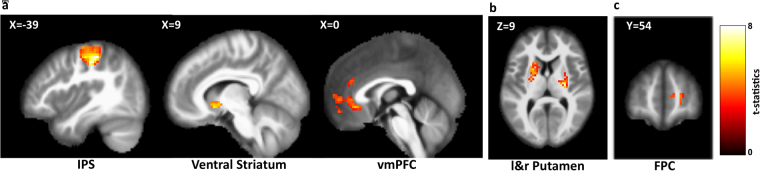
Parametric fMRI analysis. (**a**) State-action value signals were encoded in intraparietal sulcus (IPS: (−21 −36 60), k = 394, FWE corr. p < 0.05), right ventral striatum (rVS: (9 12 −9), k = 61, SVC p < 0.05)^13^, and ventro-medial prefrontal cortex (vmPFC: (0 33 −9), k = 38, SVC p < 0.05)^1^. (**b**) Reward prediction error signals were encoded in left and right putamen (rPutamen: (27 −15 9), k = 58, SVC p < 0.05; lPutamen: (−21 9 9), k = 74, SVC p < 0.05)^11^. (**c**) Cognitive entropy signals were encoded in frontopolar cortex (FPC: (12 60 3), k = 20, SVC p < 0.05)^15^. FWE: whole-brain familywise error correction. SVC: small volume correction, equal threshold with p < 0.05 and k > 10 voxels of correction within 10 mm sphere centred by known peak-coordinate was applied.

We identified signals encoding the participant’s internal value for the choice of a given stimulus in intraparietal sulcus (IPS, [−21 −36 60], 671 voxels, FWE corr. p < 0.05)^1,3,28,29^, which is widely known to encode value signals in motion-related learning processes. We then found that the value signals were encoded in the predefined areas: The vmPFC [0 33–9], 38 voxels, SVC, p < 0.05)^1,10^ and the right VS [9 12–9], 61 voxels, SVC, p < 0.05)^13^. These results successfully replicated previous findings regarding value encoding in the brain and verified the validity of our computational model (Fig. 5a, Table 2). We also examined reward prediction error signals encoded in the predefined putamen ROI (left [−27 9 9], 74 voxels, right [27 −15 9], 58 voxels, SVC, p < 0.05)^11^; activation in the ROI was correlated with the reward prediction error, consistent with the prevailing hypothesis on dopaminergic neurons^30^.

We next investigated the areas correlated with the two exploration-related signals. First, the policy entropy signal, which quantifies cognitive ambiguity, was observed to mediate exploratory behaviour in the present study. Second, the transition time-point signal, which depicted the time-points when a subject shifted his/her policy (i.e., explored a new feature). As expected, We found that policy entropy was significantly associated with activation in the FPC ([12 60 3], 20 voxels, SVC, p < 0.05)^15^. However, another exploration-related signal, the transition time-point signal, was found only with a very lenient threshold (p < 0.01 uncorrected and cluster size >7 voxels).

## Discussion

People usually learn to make choices in a situation with limited, rather than complete, information. However, few studies examined the situation of an incomplete multi-dimensional environment, especially with regard to feature exploration or learning processes^6–8,31^. In the present study, we investigated how, when, and why the exploration and learning of new features occurs by combining a probabilistic policy exploration model with a value transfer learning model based on an RL algorithm. Our subsequent fMRI analysis also revealed that the FPC encodes information pertaining to the exploration of features, providing neural correlates for the exploration of feature dimensions.

Our task, in which no instructions were provided to participants, elicited exploratory behaviour of the relevant features. Our findings demonstrated that the probabilistic policy exploration model successfully detected transitions of policy, providing trial-by-trial predictions about how features were combined to make choices. We further observed that the majority of participants tended to explore new features using their current estimation of values, rather than the previous trial’s probabilities. In addition, cognitive ambiguity (entropy) was high during exploration time-points (policy transition time points). Participants were also observed to learn new features by relating them to other known features, instead of learning each new feature independently. Thus, a participant’s behaviour was successfully explained by a value transfer learning model that utilised the learned *Q*-value as the initialisation value.

As previously mentioned, our multi-dimensional reinforcement learning task was optimized to encourage feature exploration. Most previous studies regarding reward-based decision-making have focused primarily on the stimulus itself, rather than its features^11,32,33^. Even in those studies investigating stimulus constructions, a full explanation of the stimulus’ features were provided to participants^6–8,34,35^. For instance, Niv *et al*.^6,34^ provided complete information regarding the features and confirmed participants’ awareness of these features in practice trials. Furthermore, their task was designed to investigate the attention process during dimensional reduction, rather than the exploration of new features. Our experiment included simple fixed reward probabilities to ensure participants to learn the task, and the results indicated that the task was useful for evaluating exploration behaviour with regard to several different features.

To estimate the tendency and timing of the feature exploration process, we implemented a two-stage model involving a basic RL algorithm, rather than a function approximation method. As the dimensionality of the feature space changes over time, an approximate RL requires that the parameters be reset and retrained whenever the model predicts that the association between rewards and a subset of features has changed. Moreover, approximate RL models require a fairly large number of trials relative to that required for human experiments, and it is often difficult to determine what the function approximator has learned. Although a few deep learning models utilise attention maps^36,37^, these models require a large number of training samples and are thus inapplicable for our analyses.

Therefore, we applied two probabilistic policy exploration models with a simple RL algorithm. The possible feature combinations were abstracted as seven distinct state-spaces and policies were made to choose the state-spaces, as opposed to simply assuming a reduction in feature dimensions as learning proceeds^6,35^. Observations on these policies, rather than a certain feature^7^, were modelled based on an HMM and a *softmax* function. Subsequently, the *softmax* function-based policy search model, which assumes temporal independence among policy probabilities and infers policy probabilities using state-action values, explained participants’ behaviour more precisely than the HMM-based model, which infers policy probabilities based on probabilities from the previous trial. Policy probabilities underwent fifth polynomial regression fitting to reduce transition noises before selecting the policy. This opened a possibility for HMM model to have better likelihood. To maximize the overall log-likelihood value, correct estimation of chosen policy on each trial is the most critical factor. Therefore, if the subjects’ actual choice was based on the HMM strategy, albeit unlikely, then the log likelihood value for the chosen policy will essentially be greater than the one for the policy with the maximum state-action value, *softmax* function-based model.

Model comparison results between two probabilistic policy exploration models imply that state-action values of abstracted policies influence the feature exploration process more strongly than the temporal relationships among policies. Further analysis of the model behaviour suggested that exploratory behaviour increases along with policy entropy (i.e., cognitive ambiguity). In addition, our analysis of the *softmax*-based model revealed four distinct patterns of exploratory behaviour, including exploring a new dimension (2d to 3d), exploring a new dimension but then reducing dimensionality by one (3d to 2d), and no exploration (only-2d, only-3d). However, the majority of participants (15/29) tended to explore and integrate the task components during experimental trials. Thus, our results suggest that most participants tended to explore new features when cognitive ambiguity was high.

Intriguingly, we observed that when changes in policy occurred, the values of newly added dimensional components were initialised to the values that participants had previously learned, rather than to zero. To the best of our knowledge, the present study provides the first evidence of value transfer among online policies during exploration. Furthermore, our findings are consistent with the initialising strategies reported in various studies involving machine learning paradigms^26,27^. Despite the smaller beta values observed for the value-transfer learning model due to state-space changes during policy shifting (Table 1), this model best explained human exploration and learning behaviour. In addition, the positive correlation between the model’s likelihood values and performance indicates that our computational model establishes theoretical design principles for exploration algorithms that operate during situations in which incomplete information has been provided (S7 Fig. 7).

However, the exploration of new features and learning of new policy were modelled separately. This scenario may not be entirely realistic, as feature exploration and value learning often occur simultaneously. A recent study regarding option-critic architecture^38^, which is capable of learning both internal policies and termination conditions, may provide insight into combining the two steps of our model. Future studies should aim to extend the model such that cognitive entropy-based termination is incorporated into the inferred value initialisation. In addition, several working memory-related models provide a potential explanation for feature integration within the working memory framework. For instance, Nassar *et al*.^39^ suggested that chunking of visual features facilitates encoding of visual working memory, enabling effective storage and recall. These models suggest that, given a certain number of features, the feature exploration and learning processes require certain memory trade-offs. Thus, future studies involving more features should aim to investigate feature exploration and learning behaviour in this context.

Our neural findings provide evidence for the existence of exploration of feature dimensions in policy learning. We observed that when people are integrating dimensional features, value signals were encoded in the IPS, which has been associated with value encoding in motion-related learning processes^1,3,28,29^. Importantly, our findings regarding the vmPFC and rVS are also consistent with previous findings regarding value-based decision-making (vmPFC^10–12,40–42^, rVS^12,13^), confirming that our computational learning model was properly formulated to estimate value signals. The results further suggest that transferred values are also encoded in conventional dopamine-related areas associated with value encoding (vmPFC and rVS). We also identified reward prediction error signals in the putamen previously implicated in reinforcement learning^15,43,44^. Moreover, our finding regarding the mediation of exploration by signals associated with entropy is also consistent with previous findings. For example, the FPC has been reported to encode counterfactual choices^1,16,45^, which is allowing for the option to explore new possible choices. There is one caveat, however, which is that the sample size of the feature dimension-integrating group is small (N = 13). Further work will be needed with a sufficient sample size to fully examine how these brain areas interact to increase or decrease feature dimensions.

Significantly, in accordance with the findings of previous studies, our results revealed that signal associated with exploration (e.g., cognitive entropy) is expressed in the FPC. Previous researchers have reported that the FPC is responsible for the exploration of alternative actions^1^, reliability in choosing an alternative rule^3^, and the exploration of alternative rules^15^. Moreover, previous studies have revealed that transcranial stimulation of the FPC induces exploratory behaviour^2^. Taken together, these findings suggest that exploration of new feature dimensions and the corresponding policy transitions are mediated by the FPC.

In summary, we addressed at both the behavioural and neural level how, when, and why humans explore new feature dimensions to learn and improve an online policy. Our study substantially expands the conventional understanding of exploration and learning with regard to stimulus exploration^11,33,46^, different actions^1^, and different strategies^4^. Our computational model for detecting feature exploration, the characteristics (entropy) identified by the model, and the observed correlation of FPC activation with exploration-related signals enhance the current understanding of decision making during more realistic situations, in which information regarding the situation is not always complete. Our findings may thus be used to develop more advanced reinforcement learning algorithms capable of exploring in an open space.

## Methods

### Participants

Twenty-nine subjects (12 females; age range, 20–29 years; mean age, 22.4 ± 2.27; all subjects were right-handed) were recruited from the Korea Advanced Institute of Science and Technology (KAIST) society. No participant had any history of neurological/psychiatric illness. All participants received compensation in the amount of 30,000 KRW (approximately 27 USD). Two participants were excluded due to excessive head motion during imaging (i.e. >4 mm translation in any direction, >0.08° rotation in any direction). Another four participants who failed to achieve chance-level performance (final score less than 156) (p < 0.05; simulation involving 100,000 random permutations) were excluded from further fMRI analysis. Thus, fMRI data from twenty-three participants (8 females) were analysed. An additional twenty-nine participants (11 females; age range, 20–29 years; all right-handed; no history of mental illness) were recruited for further behavioural testing, without fMRI measurement. The Institutional Review Board of Korea Advanced Institute of Science and Technology (KAIST) approved the study, and all the participants provided written informed consent. Also, all experiment procedures were conducted in accordance with the IRB guidelines and relevant regulations.

### Behavioural Task

Participants performed the multi-dimensional reinforcement learning task during fMRI scanning. No instructions regarding features or feature-score interactions were provided to participants. However, participants were informed of the basic task structure, including the number of figures per trial, possible rewards from pressing each button (left or right), immediate feedback after the choice, length of the experiment (number of stages), and time limit for the response. Participants’ understanding of the task structure was verified in a single demo trial involving a different type of stimulus (cross shape with orange hexagon pattern), and no prior training sessions were conducted. The behavioural task was designed and run using Psychopy (Psychology software in Python, v1.83.04; University of Nottingham, Nottingham, UK^47^).

Figure 1a indicates the behavioural scheme of the experiment. During each trial, participants viewed one of eight visual stimuli, which were composed of three distinct dimensional features: shape, colour, and pattern. Depending on their combinations, the eight stimuli were divided into four types: all-matched, pattern non-matched, shape non-matched, and colour non-matched. Two all-matched stimuli (blue-square-vertical pattern and yellow-circle-horizontal pattern) were associated with a reward of +10 points. Pattern non-matched combination from the all-matched group were associated with a reward of −10 points. All other combinations (shape non-matched and colour non-matched) were randomly associated with +10 or −10 points at a rate of 50% each (Fig. 1b). Points were awarded when the right thumb button was pressed. Pressing the left thumb button resulted in a “pass” (0 points).

Participants were required to make their decisions within 4 s, following which immediate feedback was presented for a duration of 2–6 s, depending on the participant’s response time. The behavioural task was composed of eight stages consisting of 32 trials each, resulting in a total of 256 trials for each participant. All 256 trials were presented in random order.

An additional 29 participants underwent further behavioural testing. Rather than using single rule for penalising a stimulus, 10 participants performed the pattern non-matched task (same as main task), 10 performed the shape non-matched task, and the remaining nine performed the colour non-matched task (S2 Fig. 2). Two additional stages were included for these participants, resulting in a total of 320 trials overall for each participant. All trials were again presented in random order.

### Probabilistic policy exploration model

Exploration behaviour of feature dimensionality was modelled using a probabilistic policy exploration model. Based on the assumption that participants construct policies at the feature level, seven policies relying on different combinations of three features were made (Fig. 2a). We first estimated the probability of taking a certain action under each policy (choice probability) (equations (1) and (2)), following which the policy used by participants during each trial was estimated, allowing us to deduce the features used based on the estimated current policy. Accordingly, the model consisted of two main parts: action value estimation and policy exploration.

#### Naïve Reinforcement Learning

For each policy, the state-action Q-value of each stimulus was estimated using a naïve reinforcement learning algorithm (Rescorla-Wagner model, equation (1))^16,17^. Therefore, seven sets of state-action values were measured for each participant, and the learning rate α was independently fitted by minimising the negative log likelihood function (equation (3)). The choice probabilities for each decision were determined by applying the *softmax* function (equation (2))^1^, using state-action Q-values.

#### Policy Exploration Models

The policy used by a participant in each trial was inferred using two independent models: a hidden Markov model (HMM)-based policy searching model and a *softmax* function-based policy searching model. The HMM-based model assumed the temporal dependency on policy probabilities, while the *softmax*-based model assumed temporal independency. Uncertainty measures were included in these two models. The HMM model utilised previous policy probabilities and transition probabilities among policies to account for uncertainty, while the softmax-based model utilised a free parameter for to account for with regard to choosing a certain policy (see Supplementary Methods: Two Policy Exploration Model). These two models were fitted with each participant’s behavioural results.

### Functional imaging and fMRI pre-processing

Functional and structural MR images were collected using a 3 T MR scanner (Siemens Magnetom Verio, Germany) at KAIST fMRI Center using a 32-channel head coil. Blood-oxygenation-level-dependent (BOLD) signals were acquired using a gradient-echo echo planar imaging (GE-EPI) sequence. Thirty-six axial slices with interleaved-ascending order were acquired using the following imaging parameters: repetition time (TR), 2,000 ms; echo time (TE), 30 ms; slice thickness, 3 mm; field of view (FoV), 192 × 192 × 108 mm; flip angle (FA), 90°; voxel size, 3 × 3 × 3 mm. The volumes were recorded at an orientation of 30° to the anterior-posterior commissure line. After one functional session, a whole-brain high-resolution T1-weighted structural image was collected using three-dimensional magnetisation-prepared rapid acquisition gradient-echo (3D-MPRAGE) sequence (TR, 1,800 ms; TE, 2.52 ms; 176 sagittal slices; FoV, 256 × 256 × 176 mm; FA, 9°; voxel size, 1 × 1 × 1 mm).

All pre-processing procedures and further data analysis were performed using Statistical Parametric Mapping software (SPM12; Wellcome Trust Centre for Neuroimaging, London, UK) and custom codes written in MatLab R2015b (The MathWorks, Inc., Natick, Massachusetts, United States). Pre-processing of functional images was performed in accordance with canonical procedures, including slice timing correction, motion correction (spatial realignment to the first image), co-registration of structural and functional images (transformation of the structural image to the mean of functional image), normalization to the Montreal Neurological Institute (MNI) template for facilitation of group analysis, and spatial smoothing with an 8 × 8 × 8 mm full-width at half-maximum (FWHM) Gaussian kernel.

### General linear model (GLM) analysis of functional images

We analysed fMRI data from 23 participants, following the exclusion of two participants with excessive head motion and four with low task performance (under the chance level score). Model based analysis was conducted along with 128 seconds high-pass filtering. The GLM contained four regressors of interest and several regressors of no interest. Four parametric regressors were extracted from the computational models and included in the regressors of interest: (1) state-action value signals at the stimulus-onset time-points for the 256 trials of the value transfer learning model; (2) reward prediction error signals for right-hand choice trials at the feedback onset time-points in the value transfer learning model; (3) cognitive ambiguity (entropy) signals at the stimulus onset time-points for the 256 trials of the softmax function-based policy search model; (4) time-points of policy transition based on stimulus onset in the softmax function-based policy search model. The regressors of no interest were: (1) stimulus onset time-points; (2) each participant’s response time at stimulus onset time-points; (3) feedback onset time-points; (4) six head-motion regressors determined in the pre-processing phase. GLM was convolved with a canonical hemodynamic response function (HRF) prior to regression analysis. The motion regressors were not convolved with the HRF function.

The GLM with HRF convolution was regressed to the fMRI data (830 scans). The first five scans were excluded to avoid T1 equilibrium effects. The estimated coefficient maps for four distinct regressors from each participant were used to compute second—level group statistics. Related brain regions were identified by testing each regressor with an ROI analysis. For an ROI analysis we performed SVC using combined ROI masks (refer to Table 2 for the results); each ROI is defined as a 10 mm sphere. We also conducted the whole-brain analysis by setting the initial voxelwise threshold to p < 0.001 uncorrected and then using FWE correction at the cluster level (p < 0.05, k > 10; refer to S12 Table 4 for the results).

### Data availability

All data, including behavioural data and fMRI results, are available at the following link: ibrain.kaist.ac.kr/software.

## Electronic supplementary material

Supplementary Information
