# Supplementary material for: Exploring Feature Dimensions to Learn a New Policy in an Uninformed Reinforcement Learning Task
Source: Sci Rep. 2017 Dec 15;7:17676. doi: 10.1038/s41598-017-17687-2 (PMC5732284; doi:10.1038/s41598-017-17687-2)
Supplement: Supplementary file 1 — Supplementary Information [file 41598_2017_17687_MOESM1_ESM.pdf]

Supplementary methods, figures, and Tables

Title: Exploring Feature Dimensions to Learn a New Policy in an Uninformed Reinforcement Learning Task

Authors: Oh-hyeon Choung[1,2,5], Sang Wan Lee*†[1,3,4], Yong Jeong*‡[1,2,3]

[1]Department of Bio and Brain Engineering, Korea Advanced Institute of Science and Technology, 34141 Daejeon, Republic of Korea

[2]KI for Health Science and Technology, Korea Advanced Institute of Science and Technology, 34141 Daejeon, Republic of Korea

[3]Program of Brain and Cognitive engineering, Korea Advanced Institute of Science and Technology, 34141 Daejeon, Republic of Korea

[4]KI for Artificial Intelligence, Korea Advanced Institute of Science and Technology, 34141 Daejeon, Republic of Korea

[5]Laboratory of Psychophysics, Brain Mind Institute, École Polytechnique Fédérale de Lausanne (EPFL), 1015 Lausanne, Switzerland

Contact information: † sangwan@kaist.ac.kr and ‡ yong@kaist.ac.kr

Supplementary methods

1. Policy simulation

To verify that the best strategy (optimal policy) for the designed task requires the use of all three dimensional features (shape, colour, and pattern), we created a decision agent that utilised a naïve reinforcement learning algorithm[17] to evaluate the performance of each policy.

Seven different policies (policy π_i) were modelled using combinations of three features, as follows (Fig. 2a); π_1, using shape feature (1 dim); π_2, using colour feature (1 dim); π_3, using pattern feature (1 dim); π_4, using shape and colour features (2 dim); π_5, using shape and pattern features (2 dim); π_6, using colour and pattern features (2 dim); and π_7, using all three features (shape, colour and pattern) (3 dim). Policies 1,2, and 3 thus required the use of only one feature dimensionality. Policies 4,5, and 6 required the use of combinations of two feature dimensionalities, and policy 7 used combination of all three feature dimensionalities.

The decision agent performed the task in the same manner as participants (i.e., 256 trials presented in random order). For each policy, the agent updated values for each stimulus using the naïve reinforcement learning algorithm (equation (1)) and made a decision using the *softmax* function (equation (2)). See section 3.1. (Naïve reinforcement learning) for further details.

S1 Fig. 1 demonstrates the mean final scores of 1,000 simulations. The free parameters α and β differed for the two task simulations. Consistent values of $\alpha = 0.1$ and $\beta = 1.5$ were used in the first simulation (S1 Fig. 1a), while seven different α and β values were used in the second simulation (S1 Fig. 1b). Theses seven α and β values represent the mean value of

fitted parameters from 29 subjects (S9 Table 1). When policies 1 through 4 were applied, performance was far below zero, indicating that these policies were not appropriate for the task. Although policies 5 and 6 exhibited high performance, policy 7 exhibited significantly better performance than both (paired t-test, $p < 0.001$). A dramatic difference was observed when different free parameters were adopted for each policy (S1 Fig. 1b) (paired t-test, $p < 0.001$).

2. Two Policy Exploration Models

2.1. HMM based policy search model

A participant's current policy was estimated by applying an HMM-based policy search model. HMM is used to detect hidden states that cannot be observed directly. As such, it has been widely utilised in both cognitive neuroscience[4,48] and computer science[18]. In the present study, we used a modified Baum-Welch algorithm of the HMM[18-21,49,50], which learned model parameters (transition probability between policies and probability of observing a certain action) to detect hidden states of the model and to determine the current policies used for each of the 256 trials.

The model was developed mainly by referring to the Baum-welch algorithm[50], and slight modifications were made to fit our behavioural situation. In particular, seven different policies were identified as hidden states, and the observations were subjects' behaviour, which is left choice or right choice, thus, the probability of choosing the left or right button for each policy was served as an emission probability of the policy (equation (2)). Transition probability A was trained using an expectation-maximisation (EM) algorithm[51] the probabilities of the initial values were regarded as equal (i.e., $1/7$). The model details are described below:

$$\alpha_t(i) = \alpha_{t-1}(j)a_{ji} \times b_i(o_t) \qquad \text{(supp. 1)}$$

$$\beta_t(i) = \sum_{j=1}^{n} a_{ij} b_j(o_{t+1}) \beta_{t+1}(j) \quad \text{(supp. 2)}$$

$$\gamma_t(i) = P(q_t = s_i | O, \Theta) = \frac{\alpha_t(i)\beta_t(i)}{\sum_{j=1}^{n} \alpha_t(j)\beta_t(j)} \quad \text{(supp. 3)}$$

$$\kappa_t(i,j) = \frac{\alpha_t(i) a_{ij} b_j(o_{t+1}) \beta_{t+1}(j)}{\sum_{k=1}^{m} \sum_{l=1}^{n} \alpha_t(k) a_{kl} b_l(o_{t+1}) \beta_{t+1}(l)} \quad \text{(supp. 4)}$$

In the expectation phase (*E*), $\alpha_t(i)$ represents the forward probability of using state i at time t, $\beta_t(i)$ represents the backward probability of using state i at time t, $\gamma_t(i)$ represents the current probability of using state i at time t, $\kappa_t(i, j)$ represents the probability of using state i at time t and state j in time $t+1$.

$$a_{ij}^{\text{new}} = \frac{\sum_{t=1}^{T-1} \kappa_t(i,j)}{\sum_{t=1}^{T-1} \gamma_t(i)} \quad \text{(supp. 5)}$$

In the maximisation phase(M), the transition probability *A* was updated. According to the model, the probability of using policy i was measured as $\gamma_t(i)$.

2.2. Softmax function-based policy search model

We also developed another policy search model using the softmax function (equation (supp. 6)), which uses the probability of choosing a certain action to estimate the probability of an individual policy in each trial. Unlike the HMM-based model, this model directly measures policy probability based on the choice probabilities, independent of the policy probabilities from previous trials. According to Bayesian theory[52] (equation (supp. 7)), the probability of a policy can be represented as $\mathrm{P}(\mathrm{a}|\pi_\mathrm{i})$ the probability of choosing a specific action given a particular policy (same as $\pi_i(a)$, equation (2))—for each trial.

$$Prob(\pi_i) = \frac{e^{\beta' \pi_i}}{\sum_{j=1}^{7} e^{\beta' \pi_j}}, \ \text{for } i \in [1,7] \quad \text{(supp. 6)}$$

$$\because \mathrm{Prob}(\pi_i) = P(\pi_i | a_k) = \frac{P(a_k|\pi_i)P(\pi_i)}{P(a_k)}, a_k = [0,1] \text{ for } k \in [1,256], i \in [1,7] \quad \text{(supp. 7)}$$

In this case, β' represents the inverse temperature parameter, k represents the trial number, and $\mathrm{P}(\pi_i)$ and $\mathrm{P}(a_k)$ represent the as prior probability of each policy and action respectively.

2.3. Policy transition inference

Policy probabilities for all 256 trials were estimated using the two policy search models. In order to eliminate noise and transient policy shifts, we performed fifth polynomial fitting to all seven policy probability signals, rather than averaging values within a time-window. Then, the policy with the highest polynomial fitted probability value among the seven policies was inferred as current policy for each trial (Fig. 3)[24,25]. In the present study, there were no trials with identical probabilities. However, if such as case were to occur, the policy taking more features into account was selected as the current policy.

3. Model comparison

We estimated the fitness of each of the following models with regard to each participant's behaviour using the maximising likelihood function L (equation (3)): the two policy searching models (HMM-based and *softmax*-based), and the two value transfer learning models (zero and learned value initialisation).

Maximum log-likelihood, Akaike information criterion (AIC)[22], and Bayesian information criterion (BIC)[23] (equation (supp. 8)) were calculated for all models and were used to compare the fitness of each model for each participant. All models were then compared using paired t-test.

$$AIC = 2k - 2\ln(L),\ BIC = -2 \cdot \ln(\hat{L}) + k \cdot \ln(n) \qquad \text{(supp. 8)}$$

k represent number of parameters, and n represents number of data points.

4. Additional model comparison

4.1. Simple Q learning (policy seven) with random decision noise model

Simple Q learning with random noise model was made as same as policy seven using case (equation (1), i=7). In this model, choice probability was computed using softmax function with random noise ε, which represents additional decision noise (equation (supp. 9)).

$$\pi_7(a_k) = (1-\varepsilon)\frac{e^{\beta Q a_k \pi_7}}{\sum_{j=1}^{2} e^{\beta Q a_j \pi_7}} + \frac{\varepsilon}{2} \qquad \text{(supp. 9)}$$

Here, a_k represents go or no-go choice, and ε represents randomness of the choice ($0 < \varepsilon < 1$). As a result, mean percent correct of choice, log-likelihood, AIC (p=0.0069), and BIC (0.00075) value revealed our suggested model, value transfer learning model, outperformed this policy 7 + ε model (Fig 4g, S10 Table 2).

References

48 Durstewitz, D., Vittoz, N. M., Floresco, S. B. & Seamans, J. K. Abrupt transitions between prefrontal neural ensemble states accompany behavioral transitions during rule learning. *Neuron* **66**, 438-448 (2010).

49 Baum, L. E. An equality and associated maximization technique in statistical estimation for probabilistic functions of Markov processes. *Inequalities* **3**, 1-8 (1972).

50 Rabiner, L. R. A tutorial on hidden Markov models and selected applications in speech recognition. *Proceedings of the IEEE* **77**, 257-286 (1989).

51 Dempster, A. P., Laird, N. M. & Rubin, D. B. Maximum likelihood from incomplete data via the EM algorithm. *Journal of the royal statistical society. Series B (methodological)*, 1-38 (1977).

52 Bayes, M. & Price, M. An Essay towards Solving a Problem in the Doctrine of Chances. By the Late Rev. Mr. Bayes, FRS Communicated by Mr. Price, in a Letter to John Canton, AMFRS. *Philosophical Transactions*

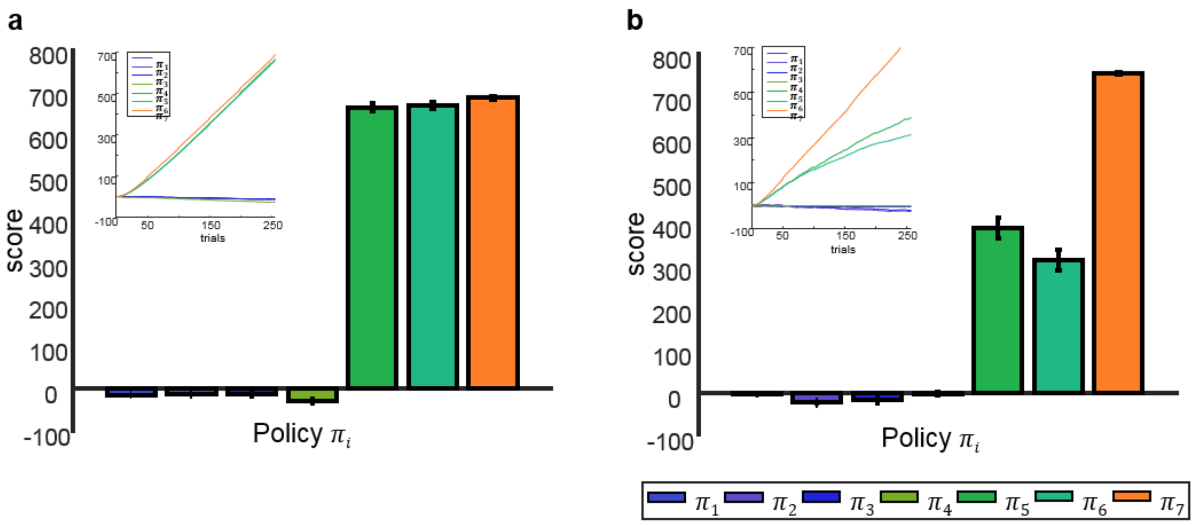

S1 Figure 1: **Policy simulation results**. Simulated score for each policy obtained after 256 trials using naïve reinforcement learning algorithm (equation (1)). Each bar represents each policy, blue: policy using one dimension, green: policy using two dimensions, orange: policy using three dimensions. **a**, Simulation with same α and β for all policies; α = 0.1, β = 1.5. **b,** Simulation with fitted α and β (average among participants) for each policy (S9 Table 1). Insets: simulated results for all 256 trials.

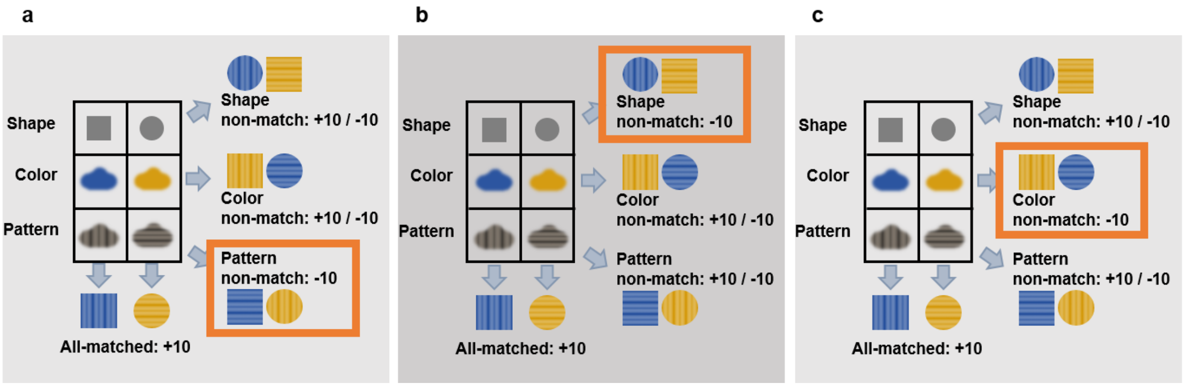

S2 Figure 2: **Additional multi-dimensional reward learning tasks.** Additional behavioural task for **a,** pattern non-matched **b,** shape non-matched **c**, and colour non-matched conditions.

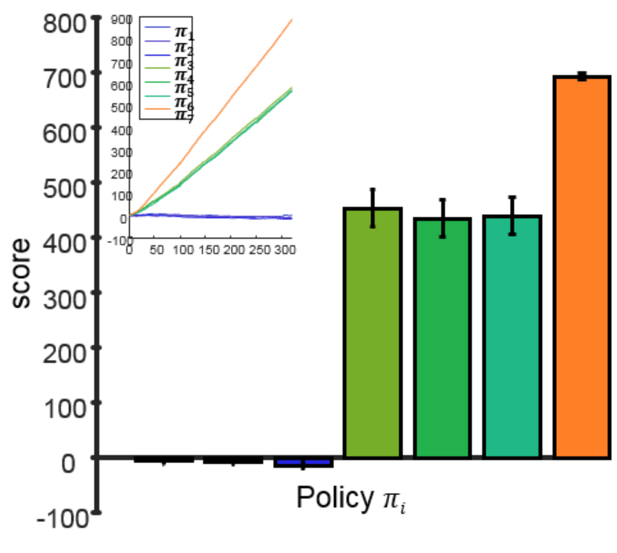

S3 Figure 3: **Policy simulation results for additional behavioural task.** Simulation results for all seven policies during the additional behavioural tasks. Identical values for parameter α and β were used in different policies as α =0.1 and β =1.5 .

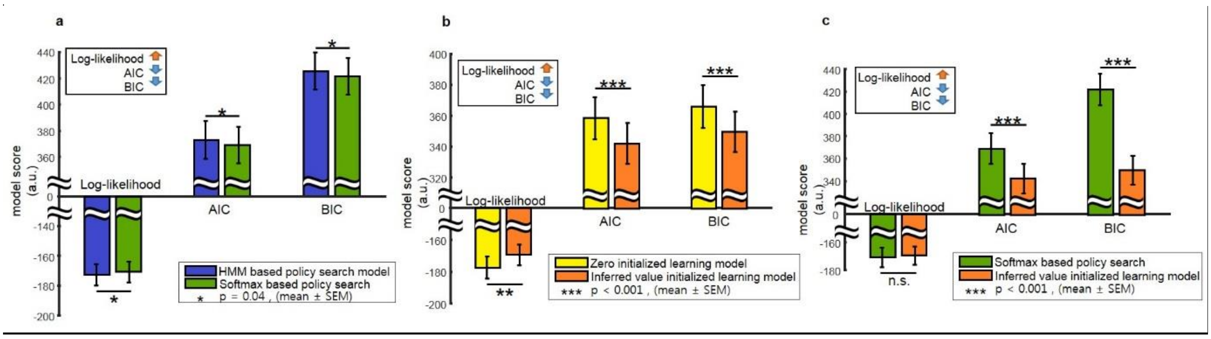

S4 Figure 4: **Model comparison for additional behavioural task**. The results were consistent with those of participants in the main task. **a,** Comparison between HMM-based policy search model and softmax-based policy search model (AIC: 373.14 ± 77.13 > 369.20 ± 75.05, BIC: 425.86 ± 77.13 > 421.91 ± 75.05, p=0.04). **b**, Comparison between zero initialised model and inferred value initialised model (AIC: 358.57 ± 74.41 > 342.18 ± 70.76, BIC: 366.11 ± 74.41 > 349.72 ± 70.76, $p=4.03*10^{-4}$). **c,** Comparison between softmax-based policy search model and inferred value initialised (value transfer learning model) (AIC: 369.20 ± 75.05 > 342.18 ± 70.76 $p=1.51*10^{-7}$, BIC: 421.91 ± 75.05 > 349.72 ± 70.76 $p=2.89*10^{-17}$). AIC: Akaike information criterion; BIC: Bayesian information criterion, mean ± SEM

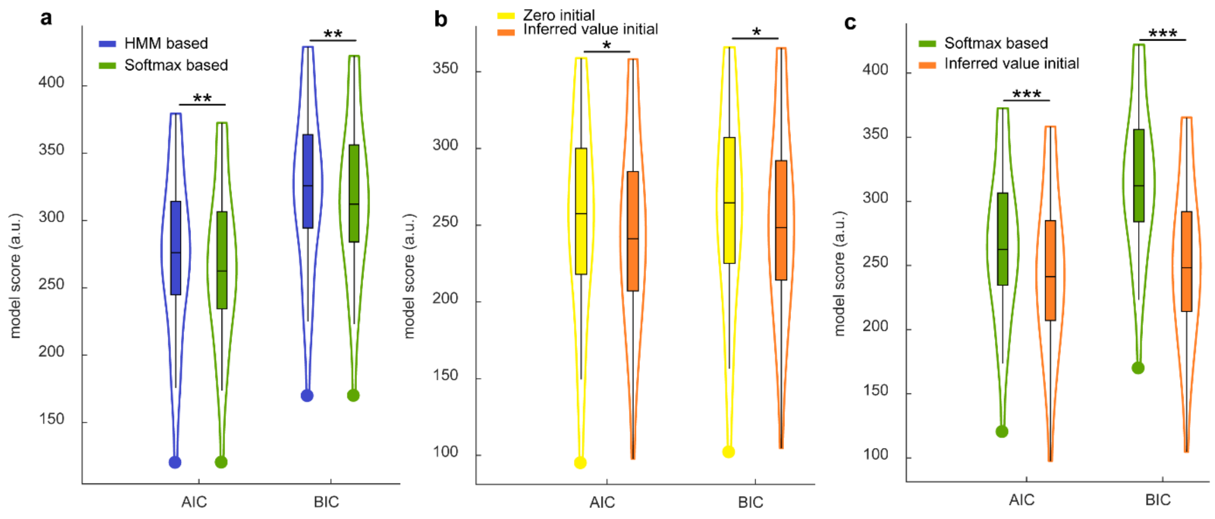

S5 Figure 5: **Model comparison results represented with violin plot**. Violin plot and inner box plot for the model comparison results. Middle line of the box plot: median, first and third lines: first and third quartiles. **a.** same as Fig 2d, **b.** same as Fig 4e, **c.** same as Fig 4f.

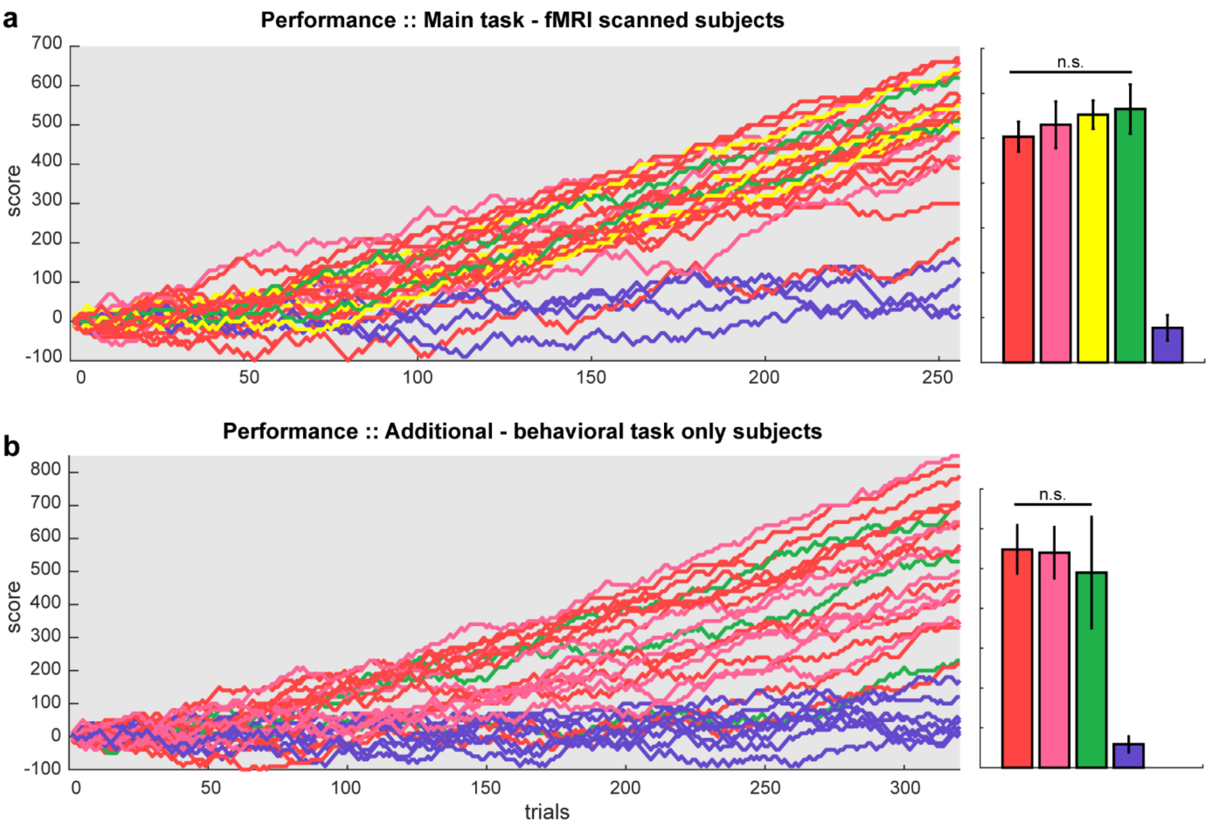

S6 Figure 6: **Behavioural performance. a,** Pattern non-matched task scores (main task). **b,** Scores for the additional behavioural task. Red: increased three dimensions group (2d to 3d); pink: increased to three dimensions but reverted to two dimensions group (3d to 2d); yellow: used three dimensions throughout the experiment (only-3d); green: used two dimensions throughout the experiment (only-2d); purple: non-learners

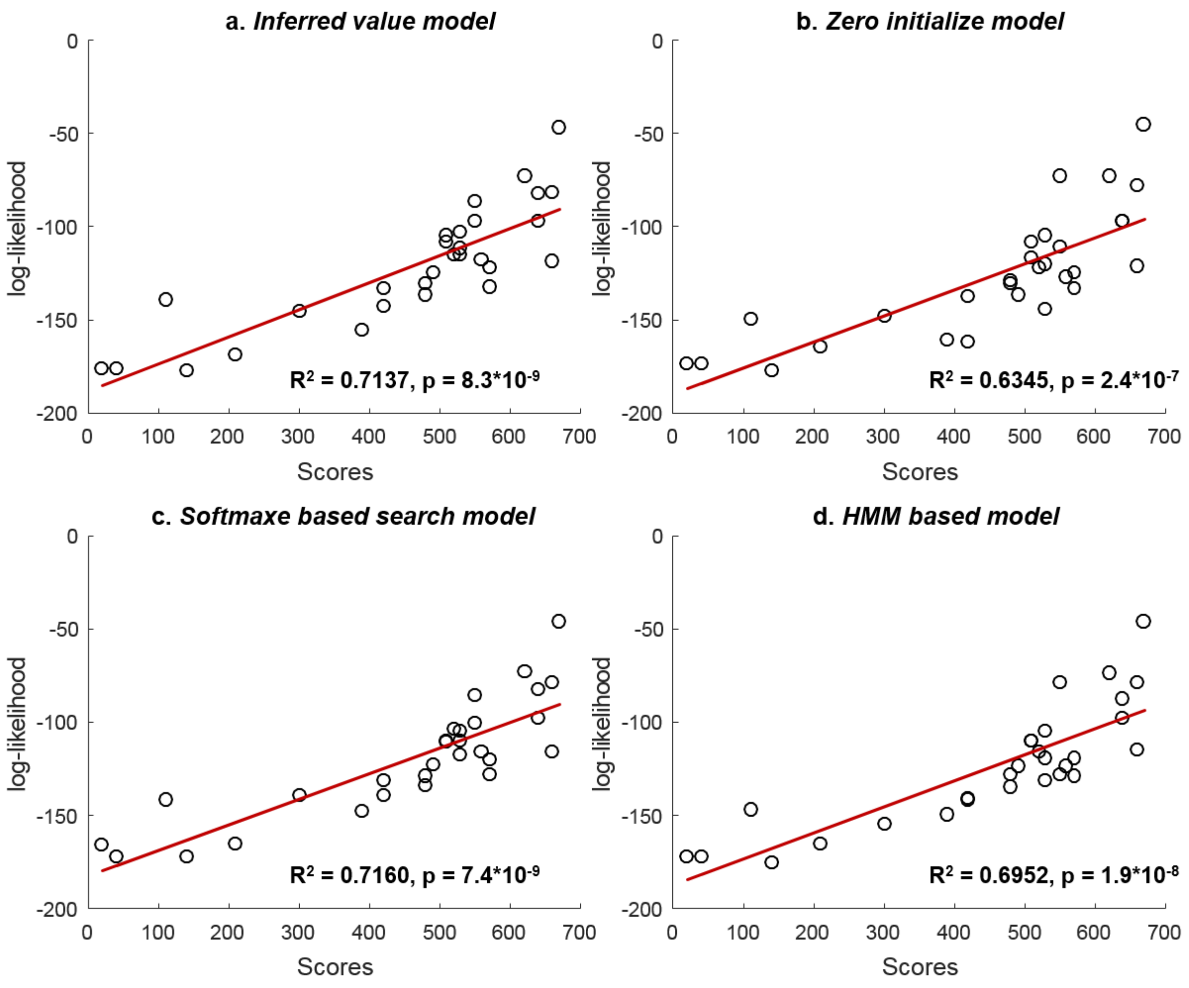

S7 Figure 7: **Model likelihood—behavioural performance relationship.** Linear regression analysis revealed a positive linear association between each participant's choice probability and behavioural performance. Significant positive linear associations were observed for all four suggested models ($p < 1x10^{-6}$). **c,** The softmax function-based policy search model, which was unrealistic but sub-optimal model, exhibited the most significant relationship ($R^2 = 0.7160$, $p = 7.4x10^{-9}$) with performance, followed by **a,** the realistic near-optimal value transfer learning model ($R2 = 0.7137$, $p = 8.3x10\text{-}9$). b,d, Significant associations were also observed for the zero-inferred model ($R^2 = 0.6345$, $p = 2.4x10^{-7}$) and the HMM-based policy search model ($R^2 = 0.6952$, $p = 1.9x10^{-8}$). HMM: hidden Markov model.

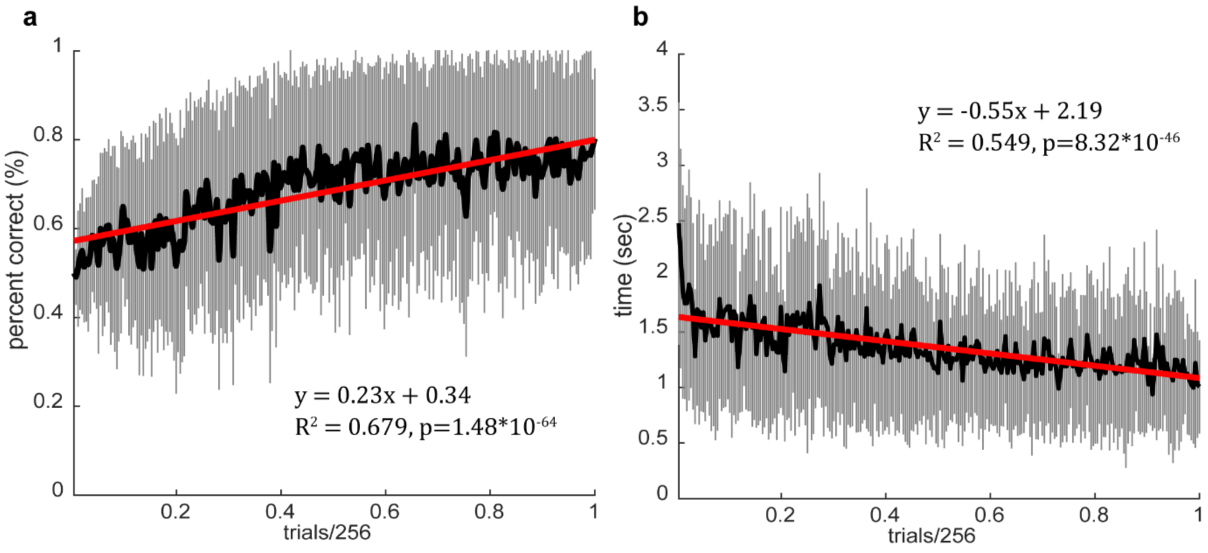

S8 Figure 8: **Model performance and participant reaction time by trial**. **a**. Percentage of correct choices for the inferred value-transfer learning model. The mean percentage pf correct choice exhibits a significant linear increase along with trial order (R^2=0.679, p=1.48*10^{-64}). **b**. Participant response times. Mean response times exhibit a significant linear decrease along with trial order (R^2=0.549, p=8.32*10^{-46}). In both panels **a** and **b**, the black line represents the mean value of all participants, while grey lines represent the mean ± SD, and red lines represent the linear fit.

S9 Table 1: **Table of fitted α and β**

	π_1	π_2	π_3	π_4	π_5	π_6	π_7
α	0.03±0.03	0.04±0.08	0.03±0.04	0.41±0.40	0.25±0.29	0.22±0.23	0.19±0.29
β	37.90±121.69	1.30±2.23	2.25±5.91	7.71±26.28	3.20±4.38	14.63±59.74	22.53±46.35

Values are represented as the mean ± SD. $\alpha < 10e^{-4}$ and $\beta > 700$ were discarded for proper estimation of average free parameters.

S10 Table 2: **Additional model comparison results**

	Log-likelihood	AIC	BIC	# of param
Policy 7+ ε	-126.31±30.31	258.63±60.61	269.26±60.61	3
Value transfer learning	-121.35±32.05	246.70±64.10	253.79±64.10	2

Table of additional model comparisons. Policy 7 + ε: simple Q-learning model with random decision noise. Value transfer learning: Our suggested model, inferred value initialised model.

S11 Table 3: **Predefined ROIs**

Signal	Brain Area	Literature			
		Coordinate	P threshold	T or Z value	Cluster size
Value	vmPFC	[-3 33 -6][1]	P_{uncorr}<0.001	Z=4.62	x
	rVS	[6 8 -4][13]	P_{uncorr}<0.001	T=7.37	5
Error	lPutamen	[-24 6 9][11]	$P_{FWE-corr}$<0.05	Z=3.51	>100
	rPutamen	[27 -13 10][11]	$P_{FWE-corr}$<0.05	Z=3.45	>100
Exploration	FPC	[18 65 10][15]	$P_{FWE-corr}$<0.05	Z=4.77	x

The ROI analysis (Table 2) was performed by small volume correction (SVC) with a combined mask of all predefined ROIs for each regressor and only clusters survived from voxel-level p-value < 0.05 was reported. We setted the initial voxelwise threshold as uncorrected p-value < 0.005 and k > 10 voxels.

S12 Table 4: **Whole brain cluster-level correction for parametric regression analysis**

Regressors		MNI Coordinate				Statistics		
		x	y	z	voxel #	Corrections	T	P_{corr}
Value	IPS	-21	-36	60	394	FWE whole-brain	10.56	p=0.0000
	rVS*	9	12	-9	31	FWE whole-brain	5.80	p=0.0107
Error	PCC	0	-48	39	40	FWE whole-brain	7.96	p=0.0056
	L visual association	-27	-96	-6	28	FWE whole-brain	7.90	p=0.0326
	SPL	0	-66	42	45	FWE whole-brain	7.59	p=0.0028
	SI	-45	-36	63	192	FWE whole-brain	7.10	$p=1.03x10^{-9}$
	OFC	-6	42	-12	46	FWE whole-brain	6.66	p=0.0025
	FPC	-45	48	-6	96	FWE whole-brain	6.54	$p=7.17x10^{-6}$
	L putamen*	-30	0	-3	66	FWE whole-brain	5.75	$p=2.01x10^{-4}$
	SPL	42	-48	57	32	FWE whole-brain	5.54	p=0.0177

FWE: whole-brain family-wise error correction. PCC: posterior cingulate cortex, SPL: superior parietal lobule, SI: primary somatosensory cortex, OFC: orbitofrontal cortex

* The cluster is as same as the cluster from an ROI analysis (Table 2).

Whole brain cluster-wise correction was performed with cluster-level p-value<0.05 and k > 10 voxels proceeded by initial threshold of p<0.001 (uncorr.).
